# Effects of localmelatonin application on post-extraction sockets after third molar surgery. A pilot study

**DOI:** 10.4317/medoral.19851

**Published:** 2014-09-30

**Authors:** Carlos Cobo-Vázquez, Isabel Fernández-Tresguerres, Ricardo Ortega-Aranegui, Juan López-Quiles

**Affiliations:** 1 DDS, MS. Postgraduate Student, Department of Medicine and Oral Surgery, Faculty of Odontology, Complutense University of Madrid; 2 DDS, MD, PhD. Associate Professor, Department of Medicine and Oral Surgery, Faculty of Odontology, Complutense University of Madrid

## Abstract

Objectives: The purpose of this study was to assess the anti-inflammatory, analgesic and osteogenic early effects of melatonin on post-extraction sockets ofpatients requiring third molars extraction. Study Design: A randomized, triple-blind clinical trial was made using a split-mouth design. Both lower third molars of 10 patients were extracted and 3 mg of local melatonin or placebo were applied. Concentrations of interleukin-6 and nitrotyrosine were determined on samples of the clot from the socket by independent ELISA tests. Radiographic bone density was evaluated by measuring Hounsfield Units in panoramic and cross sections obtained by digital scanner. Statistycal analysis by Kolmogorov-Smirnov test was performed for ELISA data. Bone density was analyzed by Shapiro-Wilk test. Subsequently t test was applied. P<0.05 was considered to be significant. Results: The concentration of interleukin-6 increased with the application of melatonin without statistically significance (361.32 ± 235.22 pg/ml vs 262.58 ± 233.92 pg/ml). Nitrotyrosine concentrations showed values below to the detectability pattern (<0.001 nM) in Optic Density curve. Bone density in panoramic sections at socket after melatonin application showed no significant difference (561.98 ± 105.92 HU vs 598.82 ± 209.03 HU). In cross sections, bone density in the alveolar region showed no significant difference(377.42 ± 125.67 HU vs 347.56 ± 97.02 HU). Conclusions: Within the limitations of this pilot study, no differences with the application of melatonin were found in terms of the concentration of interleukin-6 and bone density in post-extraction socket of retained mandibular third molars.

** Key words:**Melatonin, inflammation, pain, bone density, third molar surgery.

## Introduction

Third molar surgery is the most common intervention in oral surgery and is commonly recognized by patients as an unpleasant experience during the postoperative period (1,2). Moreover, tooth loss is a functional, aesthetic and psychological problem in patients who request for a non-removable treatment for tooth replacement without delay. This time depends on the moment that osseointegration occurs, being bone density the most important factor to take place (3-5).

Melatonin (N - acetyl-5- methoxytryptamine) is a hormone with paracrine,autocrine and endocrine antioxidant actions (6,7).

This molecule is synthesized primarily in the pineal gland to 30 years of age, time at which production decreases. It is also synthesized in other regions such as retina, gastrointestinal tract, skin, bone marrow and lymphocytes (8). One of its main physical and chemical characteristics is its high diffusion capacity since it is a highly lipophilic molecule. It also has a very high redox potential, which determines an antioxidant activity far higher than other known antioxidants (7,8). It has been demonstrated the efficacy of melatonin in the treatment of diseases caused by the presence of free radicals (7). It produces intense antiapoptotic signals even in situations of ischemia and has cytoprotective properties of enzymes involved in the healing process. It also stimulates the immune system and has oncosupresor properties (7).

The main mechanism of melatonin on surgical woundseems to be inhibition of the production of reactive oxidants, such as nitric oxide (NO) and nitrotyrosine (NT)(9), by reducing the activation of NF- kβ (10), expression of COX-2 and prostaglandin, and recruitment of polymorphonuclear cells into the site of injury (6,11,12). Moreover,melatonin reduces neutrophil infiltration and neutralizes the exacerbated production of inflammatory mediators such as necrosis tissue factor-α (NTF-α) and several interleukins (IL- 1β and IL -6) (6,13-16). It is known that the maximum antinociceptive effect of melatonin was observed 60 minutes after the systemically administration of high doses (17,12).

Regarding the bone, several studieshave reported that melatonin plays an important role in bone formation and bone metabolism (18,19). It has been shown that locally applied melatonin increases trabecular bone formation directly by stimulating osteoblasts early from endosteum (19,20), and accelerating the synthesis and mineralization of the osteoid matrix after 2 and 4 weeks (10,21). This results in a significant increase in cortical width and length of bone (20,21). It also increases bone formation markers, such as alkaline phosphatase, sialoprotein, osteopontin, and osteocalcin (22). Moreover, it interferes with osteoclast function, inhibiting bone resorption directly and by reducing RANKL (21).

Consequently, there is scientific evidence of the potential of melatonin in the treatment of inflammation and pain (6,10,23) and as a osteogenic factor that increases the volumeand bone density (20). It is also known that its effects are proportionally related to the dose applied, and there is general agreement that melatonin may be applied with insignificant adverse effects even at high doses (10,23).

Therefore, we hypothesize that local application of melatoninin the post- extraction socket produces favorable differences in the immediate postoperative period, as anti-inflammatory, analgesic and early osteogenic regarding the natural healing process of the socket.

For this set itwas aimed to determine whether there were differences in the levels of interleukin -6 and nitrotyrosine and bone density as a function of applied locally melatonin, assessing the possible complications associated with postoperative melatonin.

## Material and Methods

-Patients

Ten patients of the Department of Medicine and Oral Surgery, Faculty of Dentistry, Complutense University of Madrid indicating both lower third molar extraction, with good health (Category I and II of the American Society of Anesthesiologists [ASA]) of both sexes, aged between 16 and 35, were included. Subjects would not have a history or metabolic or systemic diseases affecting bone or healing process, no locally periodontal disease or cystyc or tumorpathology. In addition, due to standardize the sample, both third molar were in similar position and situation. Subjects who met the inclusion criteria and agree to sign the informed consent were included in the study.

Were considered as an exclusion criteria smoking, pregnant or breast-feeding, use of contraceptives or hormonal medications, the presence of chronic disorders, and history of allergy or adverse effects associated with the drugs used as well as the presence of acute inflammation or infection the day of surgery. The operation time was timed from the beginning of the incision to the end of suture, and if it exceeded 30 minutes, the patient would be excluded. Also would be excluded those who do not allow monitoring and sampling.

Both extractions were performed in the same sequence by the same operator. Melatonin (3 mg melatonin into 2 ml hydroxyethyl cellulose gel 2%) in the socket is applied randomly by computer versus placebo (2 ml of hydroxyethyl cellulose gel 2 %). In all cases the same antibiotic, antiinflammatory, analgesic and surgical intensive gastroprotective was prescribed. Healing process was revised and proceeded to suture removalat 7 days.

Patient information, treatment and samples were coded so that the side of the treatment was unknown to the patient, the surgeon and for the data analysis.

Besides the occurrence of adverse events were recorded over a time period of two months after the extraction.

-Bone density measurement

A digital cone beam scanner before surgery was carried out (Model 17-19 Next Generation i-CAT ® Imaging Sciences International, Inc. Hatfield, Pennsylvania, USA) and another at 60 days was obtained.

Bone densities were compared (i-CAT Vision program Imaging Sciences International ® version 1.8) by measuring Hounsfield Units by area (HU) using the method of quantitative subtraction radiography. In the panoramic image previous to extractions measuring 10 areas of lower third molar and 10 equal areas of bone in the region distal was performed. Measurements were made on both sides, avoiding overlapping cortical and anatomical structures. In addition, 10 cross sections of each side of the region of the lower ridge, on which five socket areas were measured, and five areas of the distal bone region were selected.

-Plasma measurements

IL-6 and NT were measured by ELISA from the clot obtained into the socket in order to quantify the anti-inflammatory effect (IL-6) and the analgesic effects (NT).

The first blood sample was obtained by direct extraction from the socket immediately after the tooth extraction. The second blood sample was obtained similarly to 60 minutes after completing the intervention.

-Statistical analysis

Data were analyzed using statistical package SPSS 21.0 for Windows (SPSS Inc., Chicago, IL, USA). Kolmogorov-Smirnov test was applied to determine the distribution of serum analysis data. Shapiro-Wilk test for bone density analysis was used. After proving to be accepted normality of samples t test was applied. The significance level was set as *P*<0.05.

## Results

Ten patients (9 females: 1 male) were studied, with a mean age of 22.60 ± 2.17 years. Samples were coded as: “a” side of melatonin prior, “b” side of control prior, “A” side melatonin later, “B” side control later. Time of surgery in any case exceed 30 minutes.

5B and 10B samples could not be analyzed by ELISA due to get as non-processable after centrifugation.

**Figure 1:**
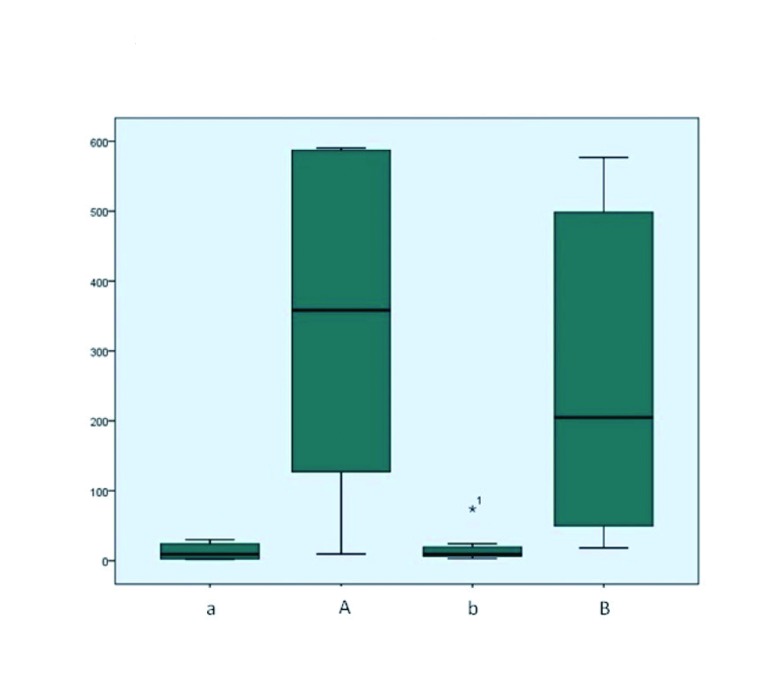
Concentrations of IL-6 (pg/ml).”a” side melatonin prior, “b” side control prior, “A” side melatoninlater, “B” side controllater. The concentration of IL-6 before treatment (11.17 ± 11.03 vs 15.03 ± 21.92 pg/ml) no significant differences (*P* = 0.517). The concentration after application of melatonin versus control (361.32 ± 235.22 vs 262.58 ± 233.92 pg/ml) no statistically significant difference (*P* = 0.465). There are significant differences in the concentration of IL-6 in advance and then to treatment with melatonin (*P* = 0.001), and with placebo (*P* = 0.026).

The concentrations of IL -6 before application of melatonin (11.17 ± 11.03 pg/ml) and placebo (15.03 ± 21.92 pg/ml)were not significantly different. After the application, there were no significant differences between melatonin (361.32 ± 235.22 pg/ml) and placebo (262.58 ± 233.92 pg/ml) in terms of the concentration of IL -6 (Fig. 1) .

Statistical analysis of the concentrations of NT could not be performed because the optical density values obtained in most of the samples were below the detectability standard curve. The lower limit of detection is set at concentrations <0.001 nM, so the NT of the samples were insignificant.

In the study of bone density at 60 days, one patient had to be excluded from the sample. Despite being excluded from the sample by radiographic study, monitoring was performed and adverse events are described.

In the panoramic sections no significant differences in bone distal region previously (a = 609.39 ± 173.28 HU; b = 620.59 ± 177.36 HU) and after extraction were found (A = 631.40 ± 91.08 HU; and B = 684.66 ± 115.50 HU). No differences in the sockets were seen between the application of melatonin (561.98 ± 105.92 HU) and placebo (598.82 ± 209.03 HU) at two months ( Table 1).

**Table 1:**
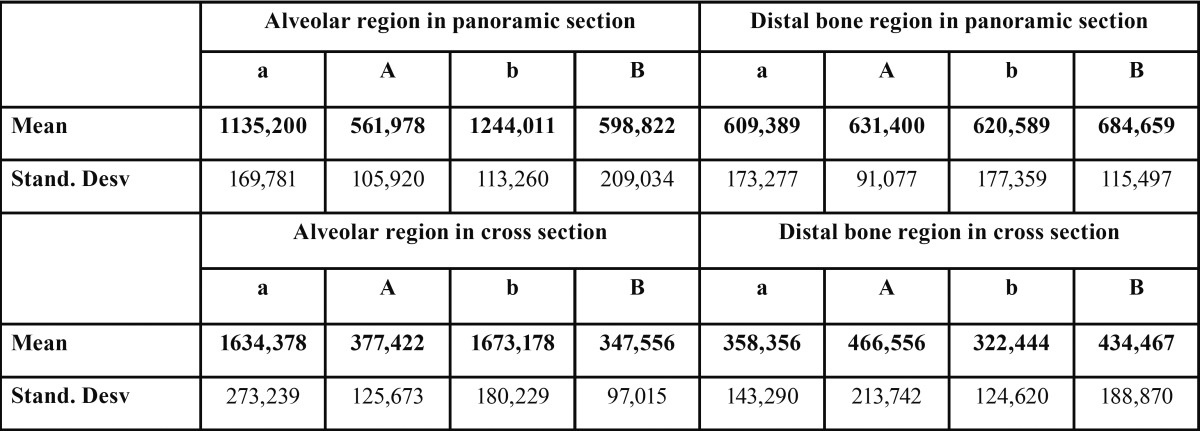
Descriptive values of bone density (UH). “UH “ Hounsfield Units, “a” side melatonin prior, “b “ side control prior, “A” side melatoni later, “B “ side controllater”. Bone density in the alveolar region prior to treatment adopts very high tailpiece being present in both the courts and the scenic cross sections values. After application of melatonin has lower bone density but without statistically significant differences from the control (*P* = 0.593) in panoramic sections, and (*P* = 0.223) in the cross sections values. Bone of the distal region, no statistically significant difference in bone density previously and subsequently to the treatment with melatonin (*P* = 0.669) on panoramic sections, and (*P* = 0.080) in the cross sections. The distal bone region no significant differences after application of melatonin or placebo in panoramic sections (*P* = 0.191) and in the cross sections (*P* = 0.391).

In the cross sections no differences in bone distal region were found before (a = 358.36 ± 143.29 HU; b = 322.44 ± 124.62 HU), and after treatment application (A = 466.56± 213.74 HU;B = 434.47 ± 188.87 HU). Bone density in the alveolar region after application of melatonin (A = 377.42 ± 125.67 HU) and placebo (B = 347.56 ± 97.02 HU) showed no significant differences.

## Discussion

This is the first work where the anti-inflammatory, analgesic and osteogenic effects of melatonin are measured after extraction of third molar in patients.

An increase in inflammation and pain have been described in surgical procedures of longer duration, so the time of surgery has been considered an indicator of difficulty (1,2). The present study is within the time of surgery recorded by other authors, between 11.03 and 25.00 minutes (2).

Despite obtaining a sufficient amount of blood for processing all samples, there were two post- placebo gel application whose plasma samples could not be analyzed as having an oily consistency. This may be due to the limited local bleeding, which mixed with the gel, resulted in an impossible processing of the sample.

Regarding the concentration of IL-6, on both sides there was a non-inflammatory state before treatment application. After treatment no significant differences in the concentration of IL-6 were found, so our study cannot claim that melatonin influences the concentration of IL-6 in relation to the early inflammatory process. A possible explanation, according to Mayo *et al.* (24), Cutando *et al.* (9) and Radogna *et al.* (6), should be that the greatest effect of melatonin occurs when inflammatory mediators are increased in the damaged region. Another factor related to its effect, according to Maldonado *et al.* (16) and Laste *et al.* (12), is the dose applied, which could be small in our study, although antiinflammatory and analgesic effects are described applying locally 2 mg (9)and even micromolar doses (6,23).

The NO has a rapid degradation of compounds such as nitrotyrosine, so determination can detect earliest and smaller changes (17,25). In most samples, values below to detectable (<0.001 nM) were obtained. Although it is not possible to draw conclusions from these findings it follows that the values of products of oxidative and nitrosative degradation immediately after extractions are not yet high enough to be quantified, according to Hamza et al. (17)in extraction of wisdom teeth, which were not detectable. Furthermore, studies such as Mayo et al. (24) support the utility of melatonin as anti-inflammatory agent but its effects would be amplified when free radicals and inflammatory mediators were already present in the injured area (17).

In the study of bone density, a patient had to be excluded from the sample due to an allergic reaction. He also received surgical treatment in the region operated due to a postoperative complication and was not possible to makethe mandibular scanner on deadline. The exclusion of this patient supposed that all the patients in the sample were women.

Bone density was obtained in panoramic sections in the distal regions (a = 609.39 ± 173.28 HU; b = 620.59 ± 177.36HU; A = 631.40 ± 91.08 HU; B = 684.66 ± 115.50 HU). This was comparable to that obtained by Hiasa *et al.* (26), Norton *et al.* (27), and Turkyilmaz *et al.* (28) in the same region (628.2±209.2HU (26), 669HU (27) and 715.8±190HU (28)), considering that bone density use to be lower in women and younger patients (29). No significant differences in bone density in the alveolar region were observed according to the treatment (a = 561.98 ± 105.92 HU;B = 598.82 ± 209.03 UH), although values are closeto the control region.

In the cross sections bone density in the alveolar regions (A = 377.42 ± 125.67 HU; B = 347.56 ± 97.02 HU) does not depend on the treatment and they were much lower than those obtained in sections of the same region in the study of González- García *et al.* (30), (524.3 ± 169.5 HU) in older patients.

Complications were recorded and the usual therapeutic approach in each case was adopted to achieve its resolution effectively. From the third day, two patients reported dizziness, which were progressively reduced until it disappears in a maximum of four days of occurrence. A lower lipdysesthesia happened on the side of melatoninand was resolved within 3 days with anti-inflammatory therapy. One patient reported fever of 39°C, discarding oral origin at examination. Another patient had a post-extraction alveolitis on the side of melatonin application. At 36 days she developed an increasingly severe noninfectious inflammation due to trauma in the periosteum, which was resolved 10 days after. Finally, one patient showed an allergic reaction to metamizol 575 mg, not mentioned in the medical record at 10 hours after surgery. At 35 days, he went to the emergencies with an increased inflammation, trismus and night pain on the side where melatonin was applied. He was diagnosed of “paramandibular abscess of 38” and treated with surgical drainage and antibiotic (ciprofloxacin 500 mg), anti-inflammatory (ibuprofen 600 mg), analgesic (paracetamol 650 mg) and an antiseptic (chlorhexidine 0.2%) therapy.

Despite the reported complications, the distribution of the samedoes not determine its relation to the application of melatonin.

## Conclusion

Finally, within the limitations of this study, we can conclude that there were no differences between the application of melatonin and placebo in terms of the concentration of interleukin-6 and bone density. No differences could be determined between nitroty-rosine levels before and after the treatment. Thus, we must conclude that the treatment is not influential. Moreover, it was not possible to establish a causal relationship of melatonin with complications occurring during the postoperative period. Further researches are necessary by applying higher doses of melatonin and extending the sample with an appropriate methodology in order to confirm these results.
